# Supplementary material for: Early intermittent hyperlipidaemia alters tissue macrophages to fuel atherosclerosis
Source: Nature. 2024 Sep 4;634(8033):457–65. doi: 10.1038/s41586-024-07993-x (PMC11464399; doi:10.1038/s41586-024-07993-x)
Supplement: Supplementary file 1 — Supplementary Methods and Supplementary Data. Description of the methods for diagnostics assessments of the Bayesian relevant life-course exposure models, as well as Supplementary Figs. 1–10, showing the corresponding results. [file 41586_2024_7993_MOESM1_ESM.pdf]

nature portfolio

https://doi.org/10.1038/s41586-024-07993-x

Supplementary information

Early intermittent hyperlipidaemia alters tissue macrophages to fuel atherosclerosis

In the format provided by the authors and unedited

Early intermittent hyperlipidaemia alters tissue macrophages to fuel atherosclerosis

Takaoka et al. Supplementary Methods

Diagnostics assessments of the Bayesian relevant life-course exposure models (BRLMs)

The assessment of diagnostics focused on convergence, mixing, parameter autocorrelation, and parameter identifiability. We utilized trace plots and R-hat values to evaluate convergence and mixing. Autocorrelation of the parameters was analyzed using autocorrelation function plots. Identifiability was assessed through pairs-plots. The effectiveness of the sampler was assessed using effective sample size Neff metrics. The diagnostics, conducted using the final fitted models (specifically model 2), indicated satisfactory convergence, mixing, and effective sample sizes. Furthermore, no issues with autocorrelation or identifiability were observed (Figure S1 for non-HDL-C and the presence of carotid plaque; Figure S2 for non-HDL-C and the presence of carotid plaque with the missing covariates by multiple imputation; Figure S3 for non-HDL-C and carotid plaque areas; and Figure S4 for non-HDL-C and carotid plaque areas with the missing covariates by multiple imputation).

Methods for imputing missing values have been described (https://github.com/MadathilSA/BayesianRelevantLifeCourseExposure/blob/master/Manuscript III/HeNCeIndiaSmkModelImput.jags). Multiple imputation was undertaken for the covariates of youth age, ever smoked, family history of cardiovascular disease, education (number of years studied), and cumulative body mass index, systolic blood pressure and

high-density cholesterol, using predictive mean matching via the R library, mice. Three imputations were made, and the means and credible intervals were calculated using the draws across all imputations.

A. Trace plot

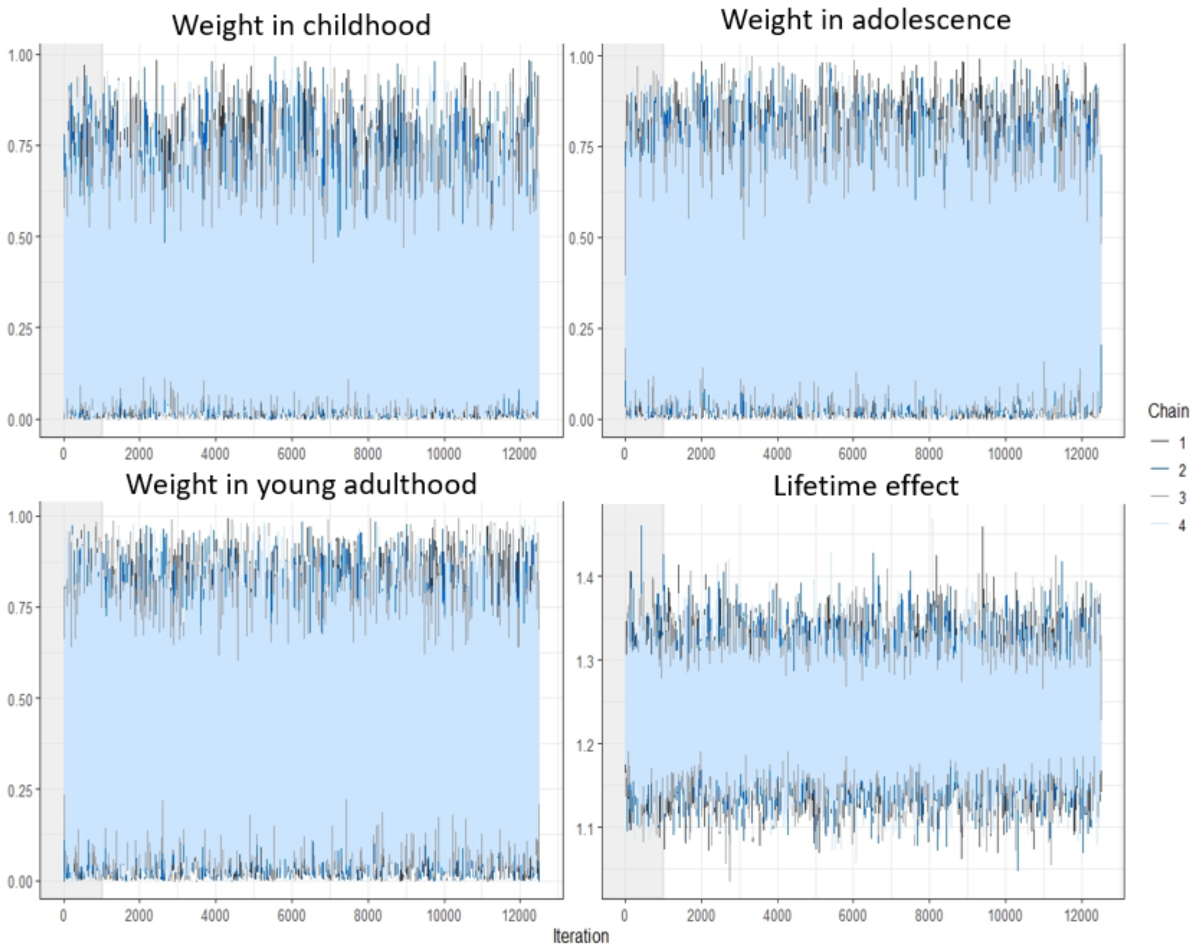
Weight in childhood
Weight in adolescence
Weight in young adulthood
Lifetime effect
Chain
1
2
3
4
Iteration

B. Rhat values

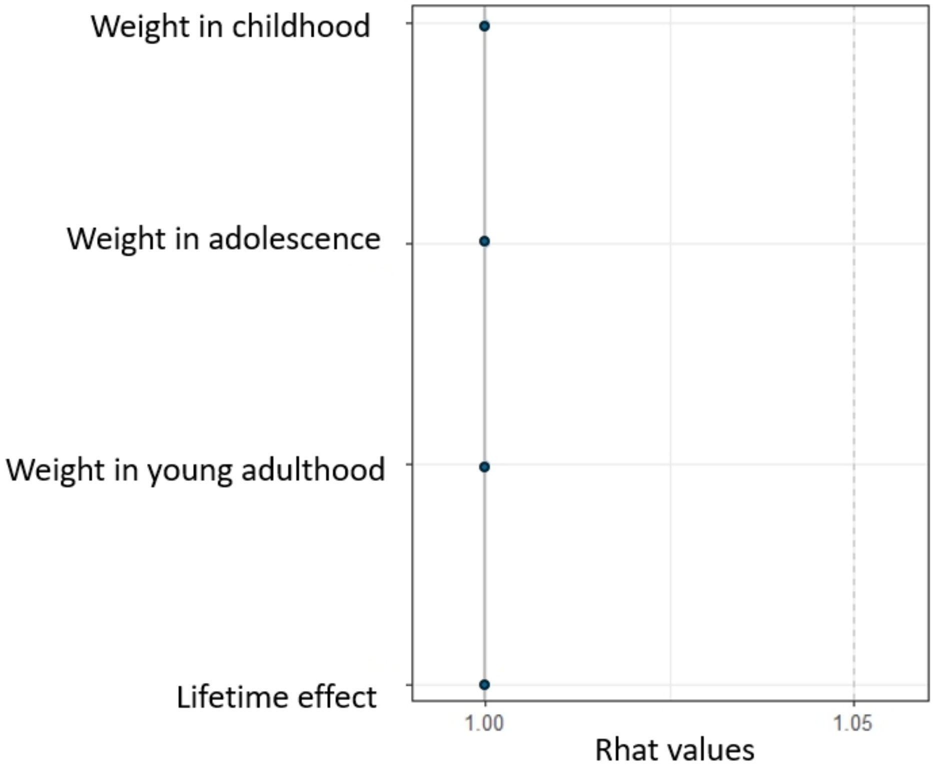

C. Effective sample size

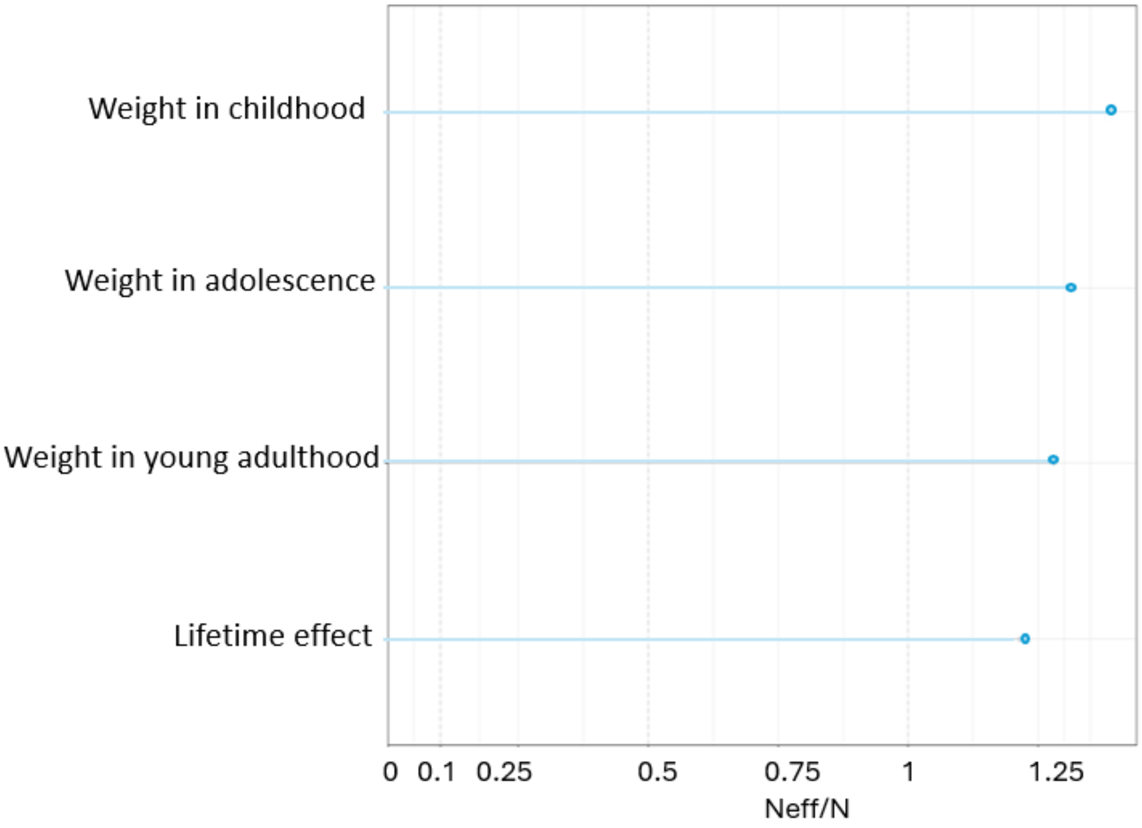

D. Autocorrelation function plot

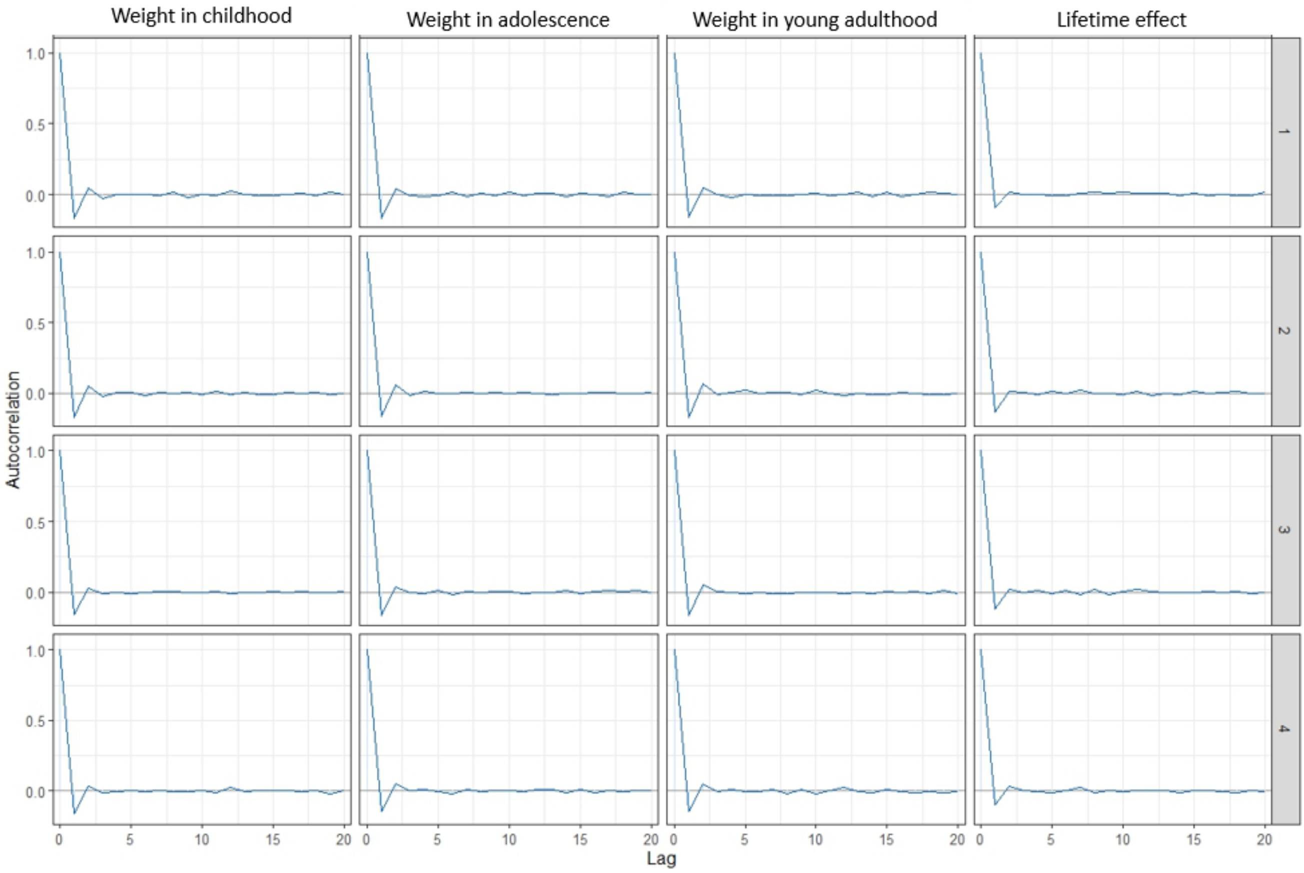

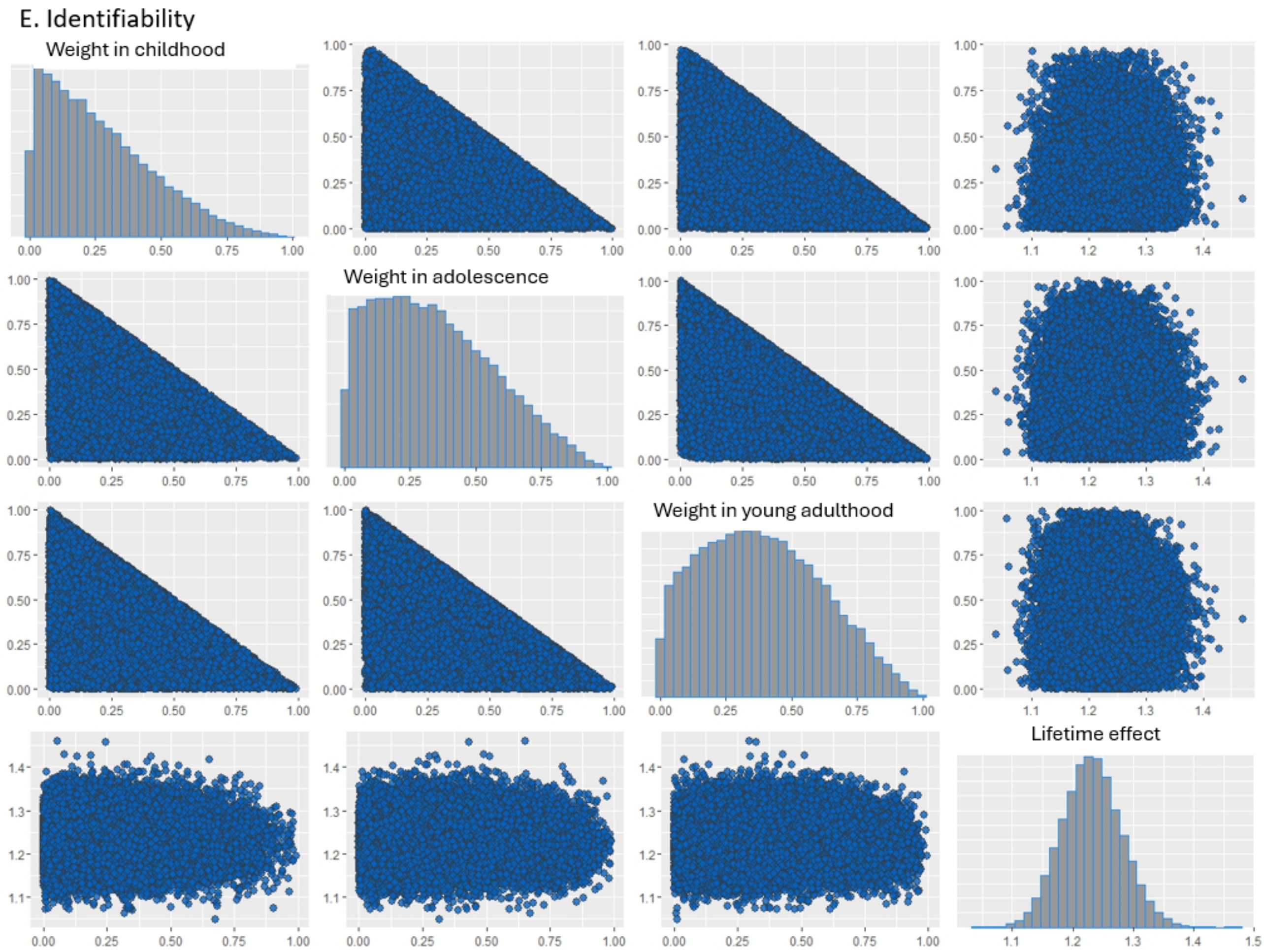

Figure S1. Diagnostics of the Bayesian relevant life-course exposure model, including (A) trace plots, (B) Rhat values, (C) effective sample size, (D) autocorrelation function plots, (E) identifiability of the parameters, for non-high-density lipoprotein cholesterol and the presence of carotid plaque in mid-adulthood aged 41 to 56 years in 2018.

A. Trace plot

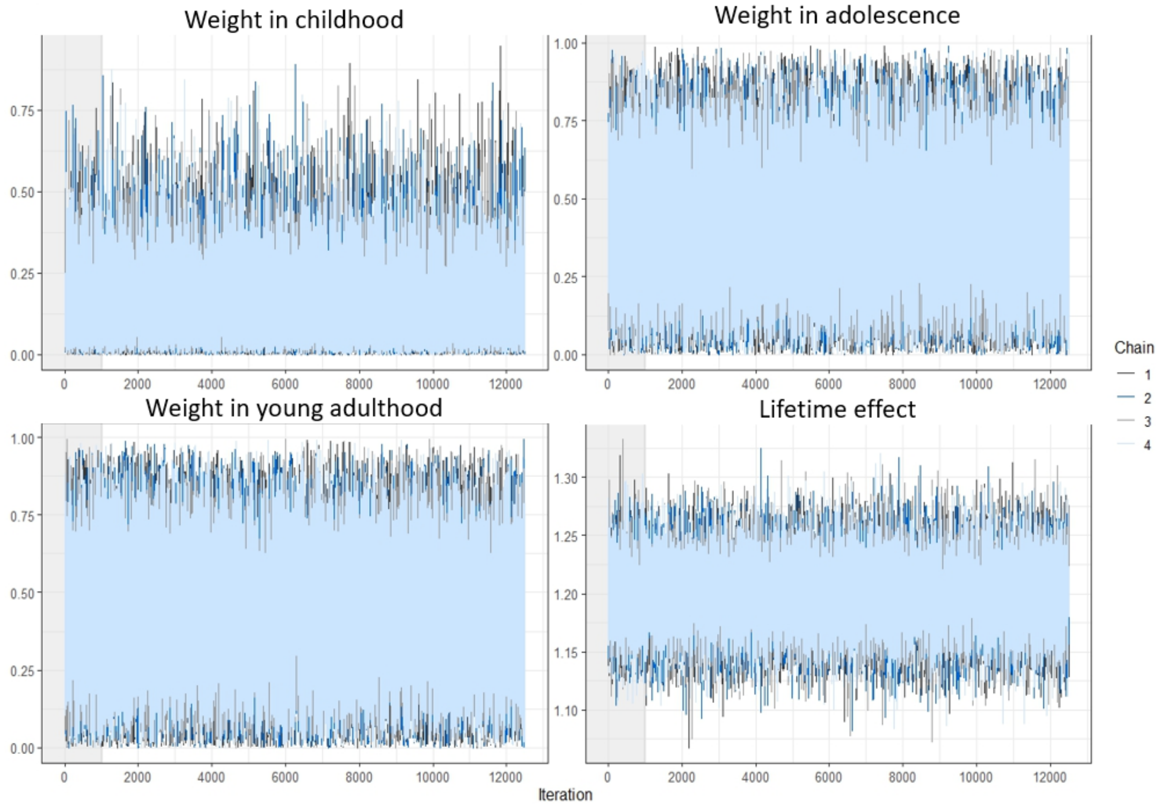

B. Rhat values

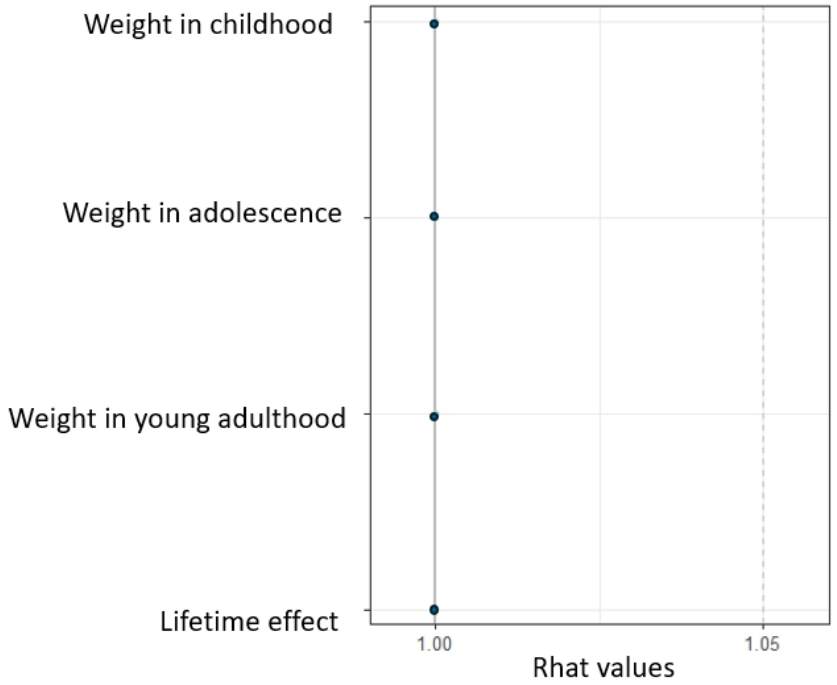

C. Effective sample size

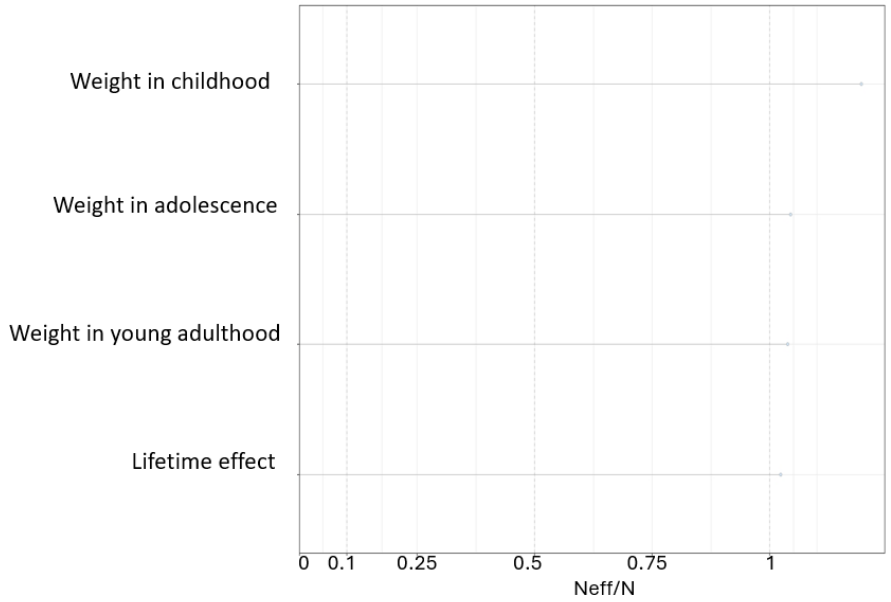

D. Autocorrelation function plot

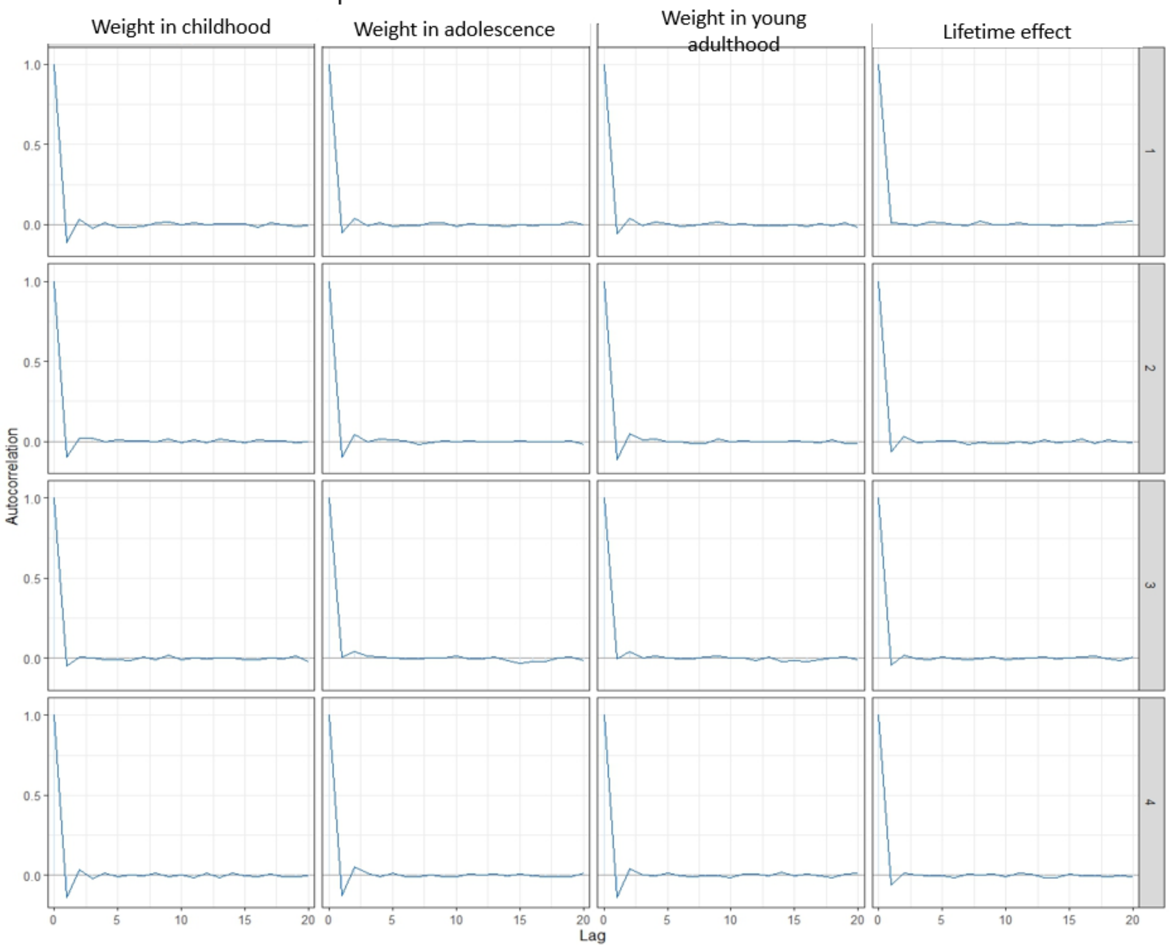

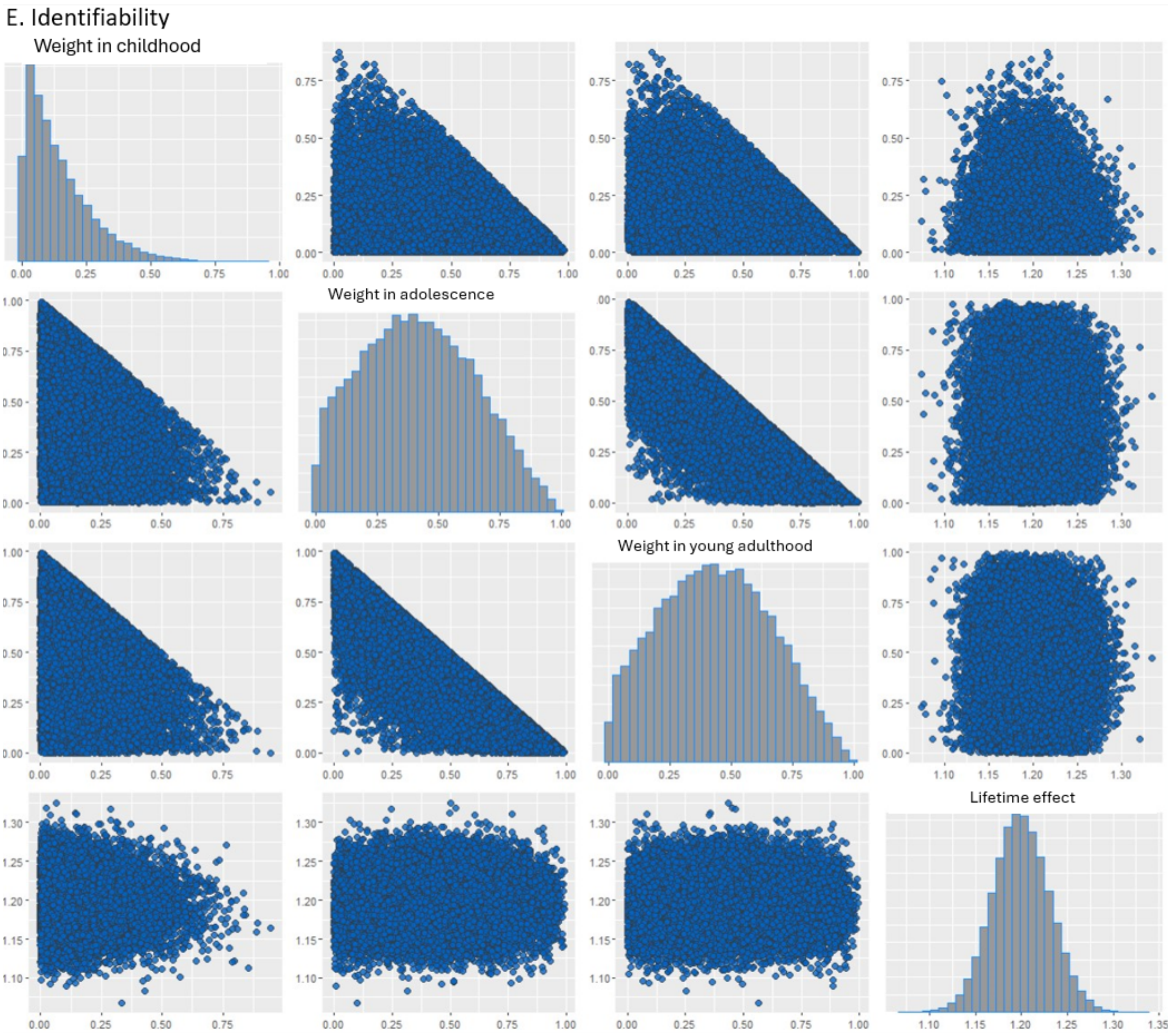

Figure S2. Diagnostics of the Bayesian relevant life-course exposure model, including (A) trace plots, (B) Rhat values, (C) effective sample size, (D) autocorrelation function plots, (E) identifiability of the parameters, for non-high-density lipoprotein cholesterol and the presence of carotid plaque in mid-adulthood aged 41 to 56 years in 2018. Missing covariates were imputed by multiple imputation, missing non-HDL-C data were interpolated by the Individual Growth Curve Model.

A. Trace plot

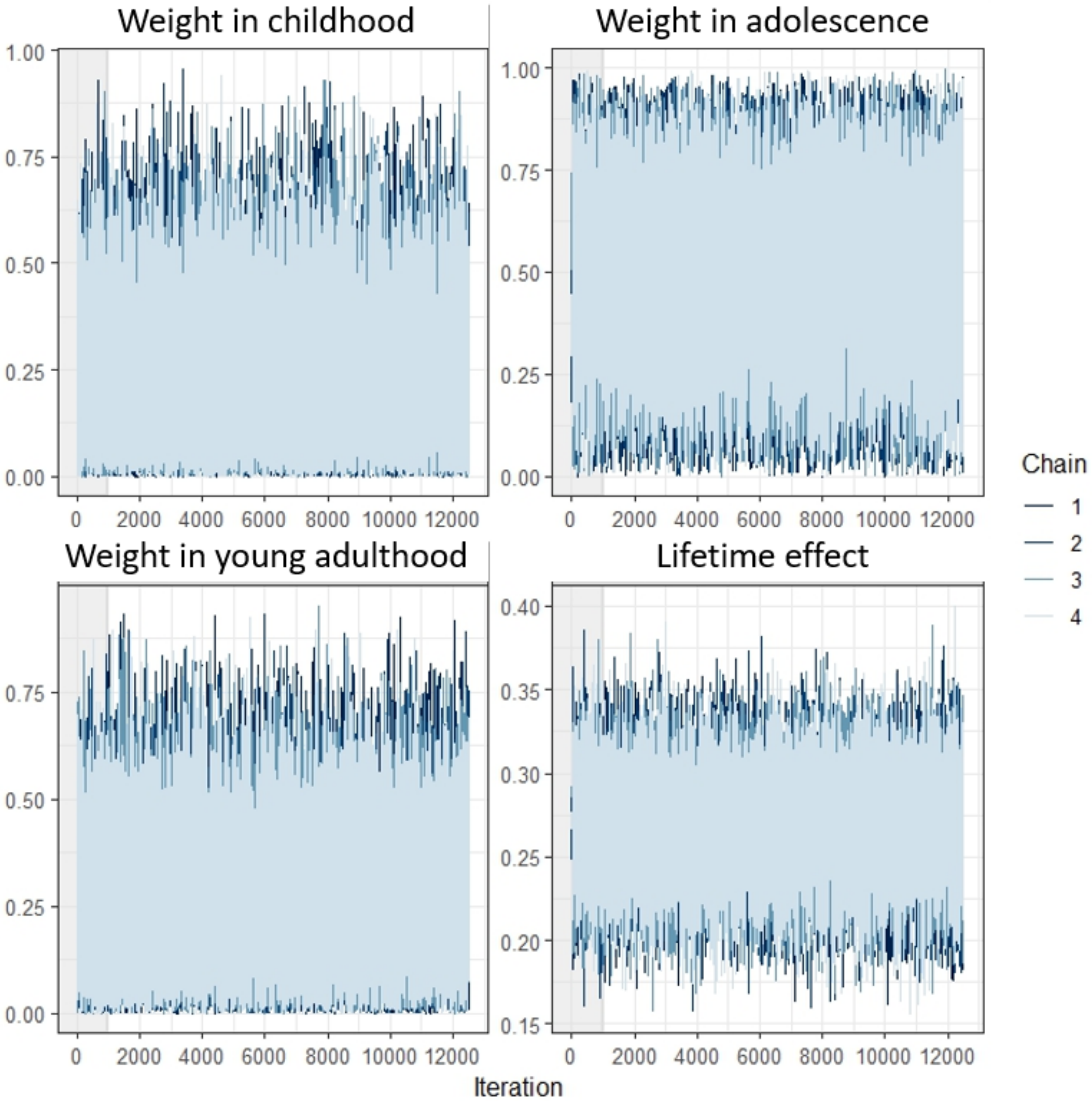

Weight in childhood
Weight in adolescence
Weight in young adulthood
Lifetime effect
1.00
0.75
0.50
0.25
0.00
0.40
0.35
0.30
0.20
0.15
0 2000 4000 6000 8000 10000 12000
Iteration
Chain
1
2
3
4

B. Rhat values

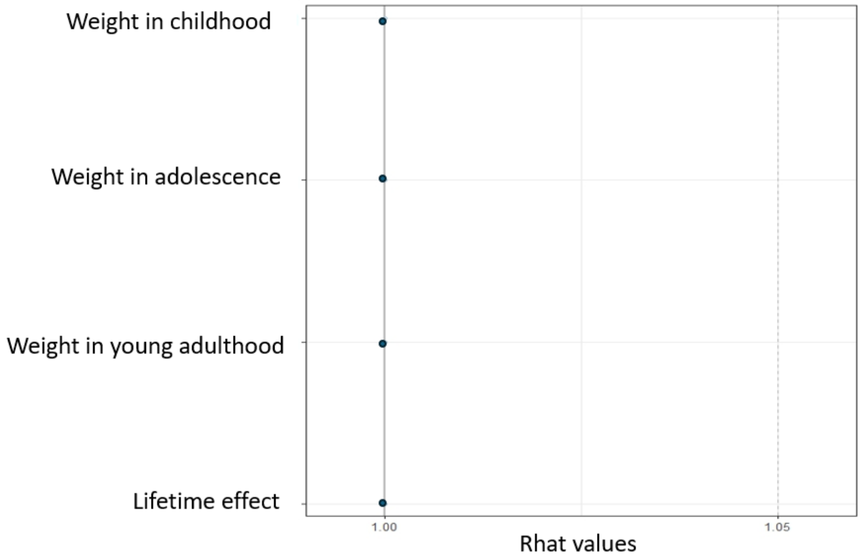

C. Effective sample size

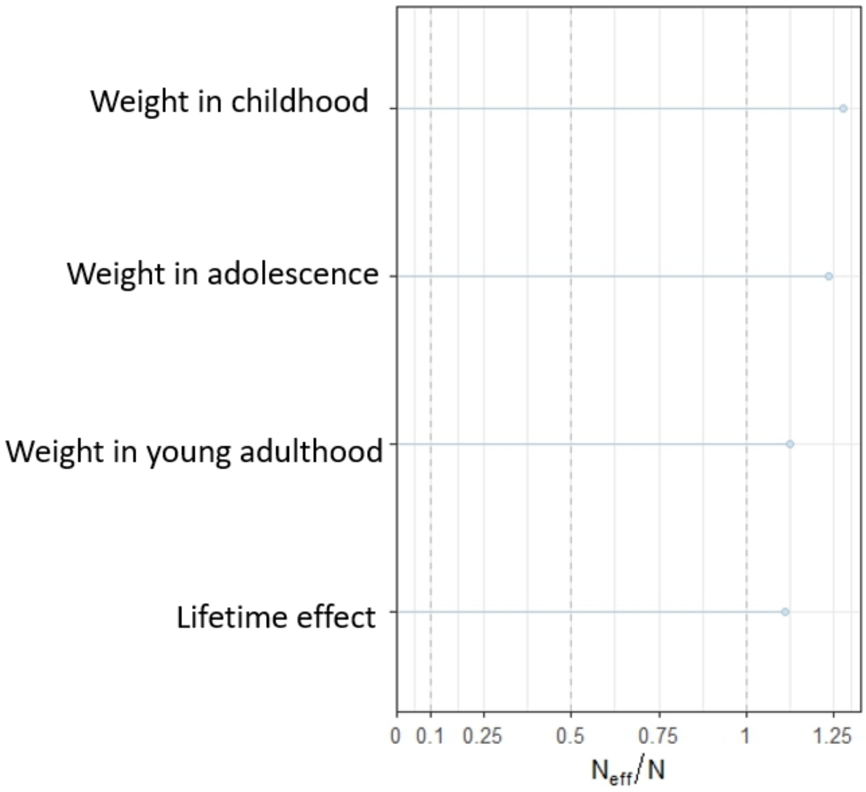

D. Autocorrelation function plot

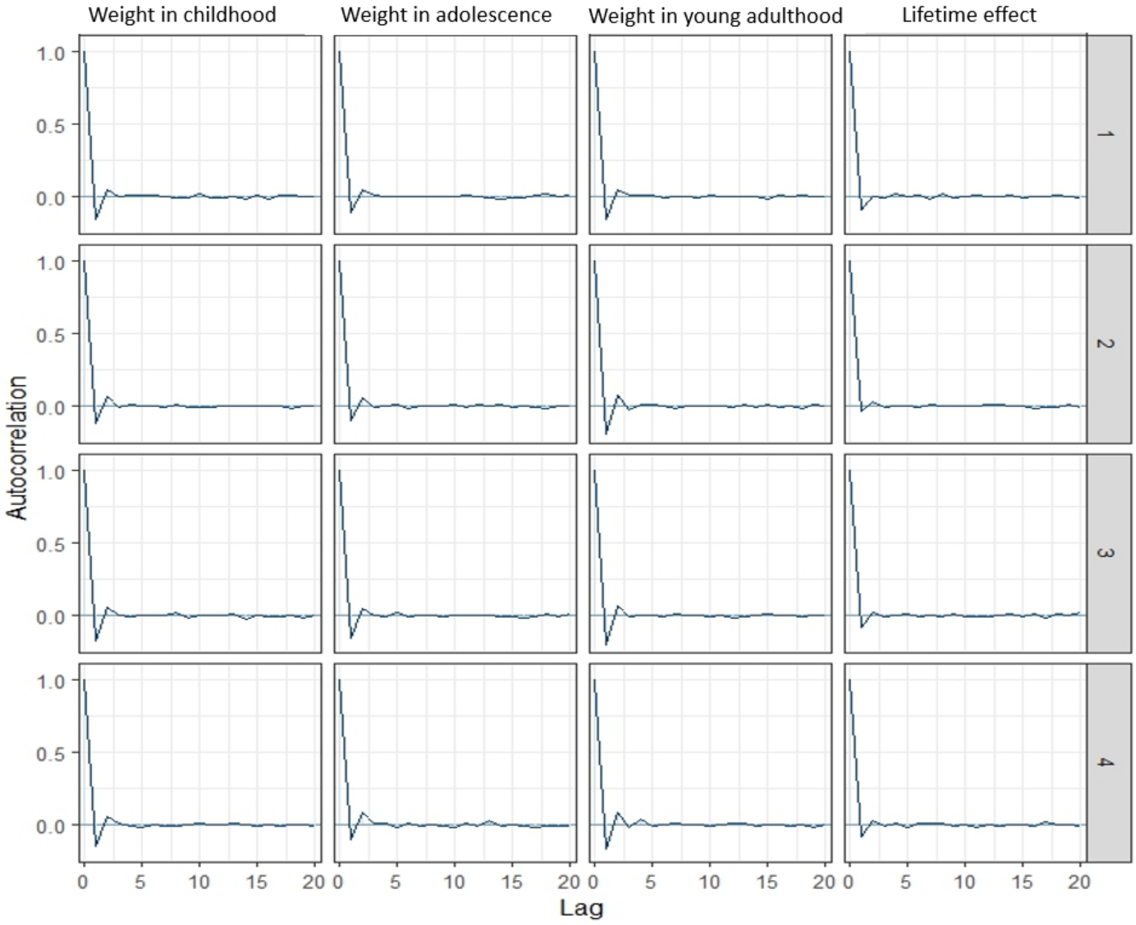

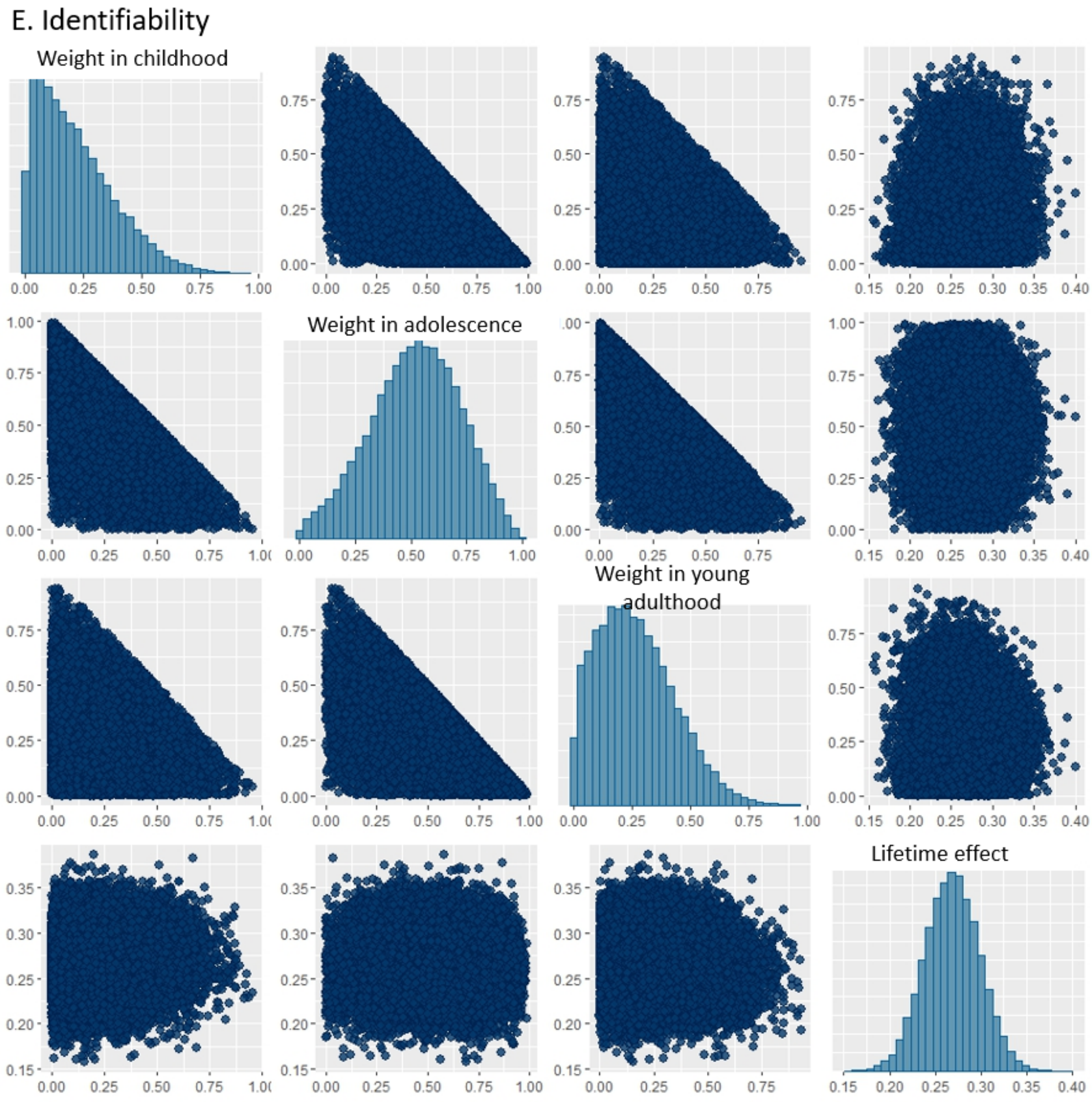

Figure S3. Diagnostics of the Bayesian relevant life-course exposure model, including (A) trace plots, (B) Rhat values, (C) effective sample size, (D) autocorrelation function plots, (E) identifiability of the parameters, for non-high-density lipoprotein cholesterol and carotid plaque areas in mid-adulthood aged 41 to 56 years in 2018.

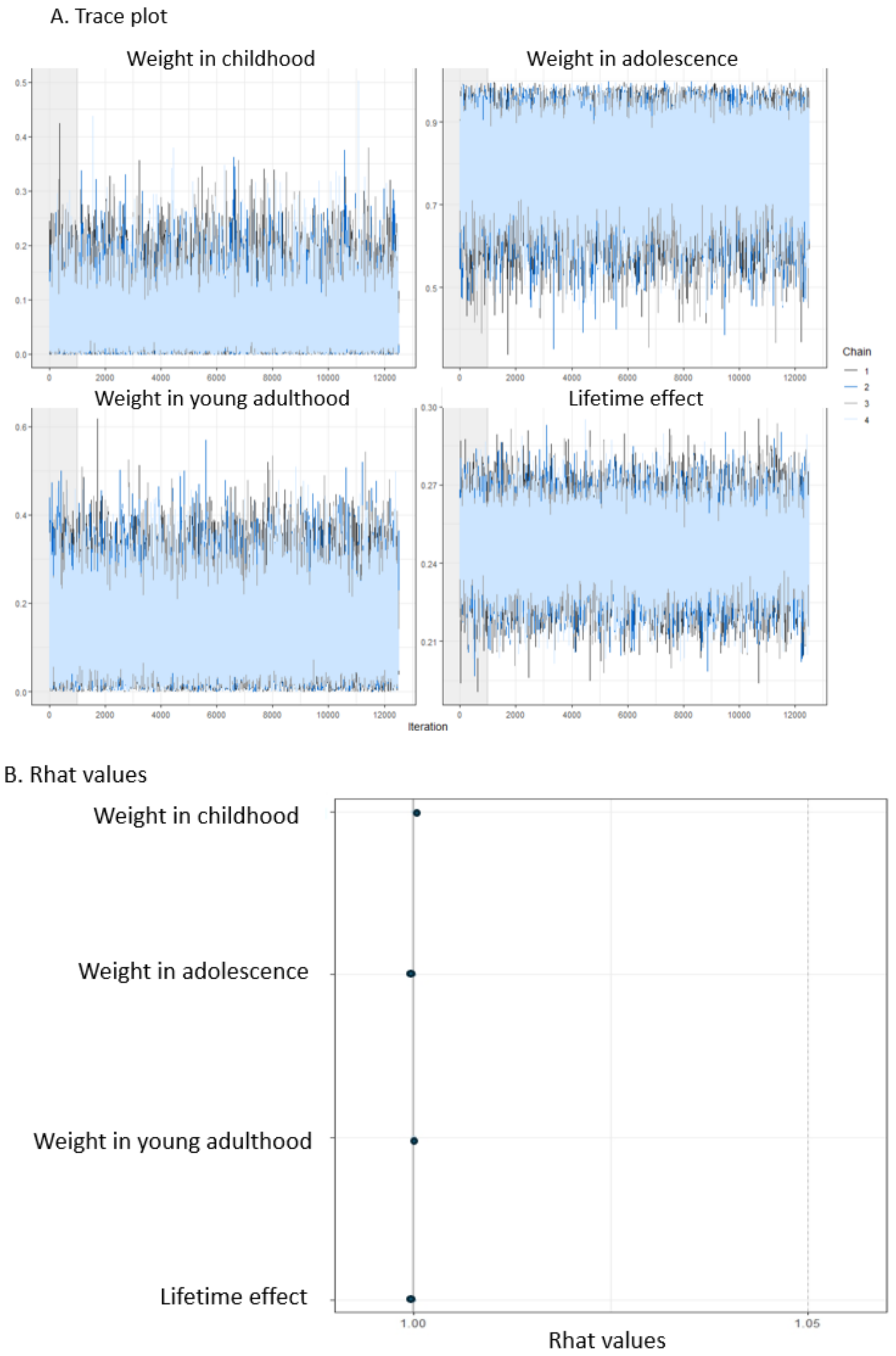
A. Trace plot
Weight in childhood
Weight in adolescence
Weight in young adulthood
Lifetime effect
Chain
1
2
3
4
Iteration
B. Rhat values
Weight in childhood
Weight in adolescence
Weight in young adulthood
Lifetime effect
1.00
1.05
Rhat values

C. Effective sample size

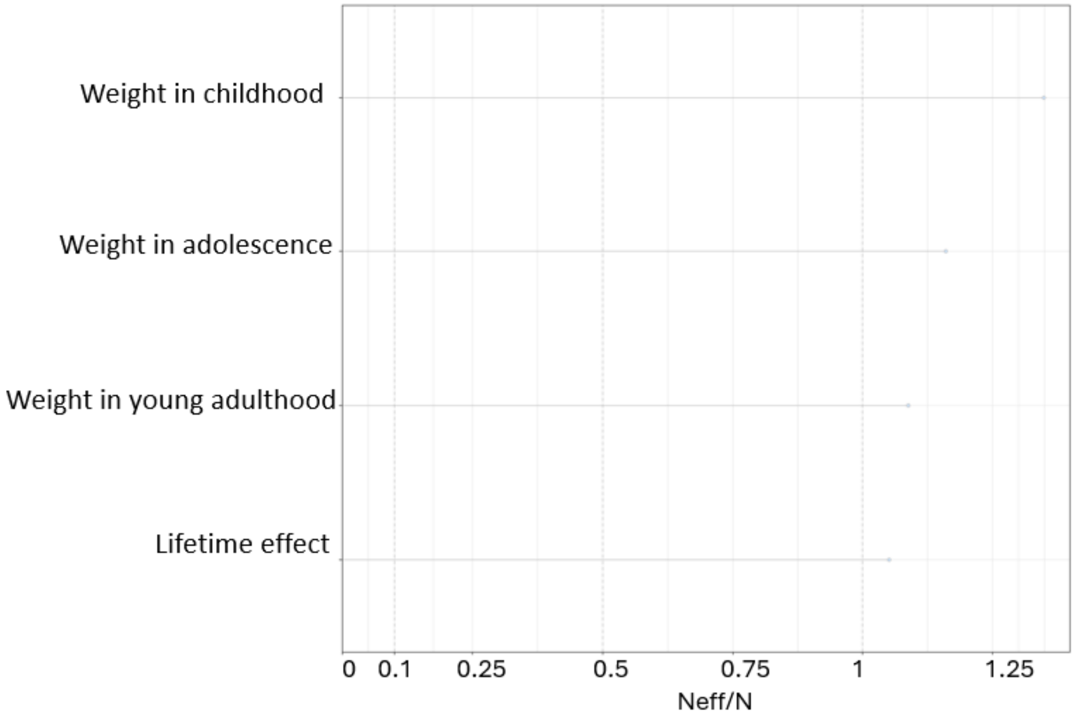

D. Autocorrelation function plot

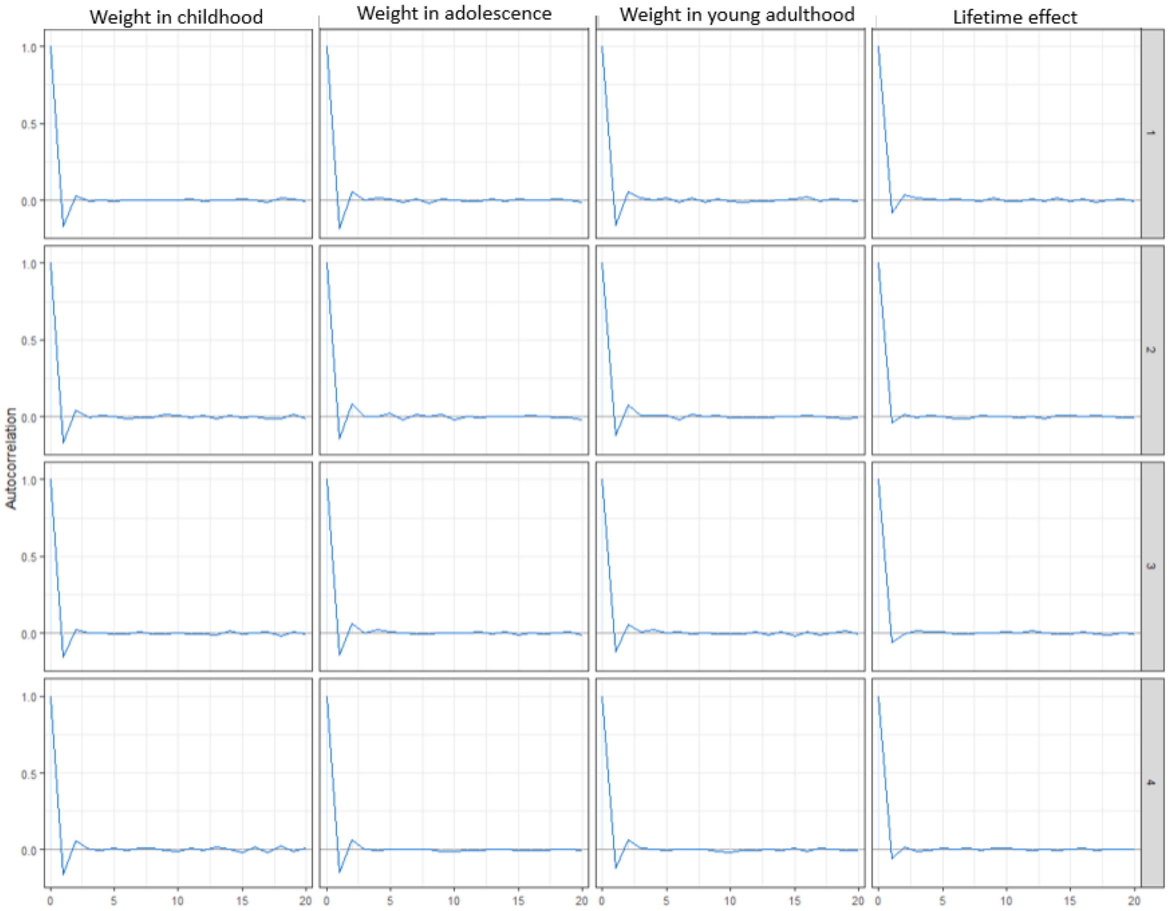

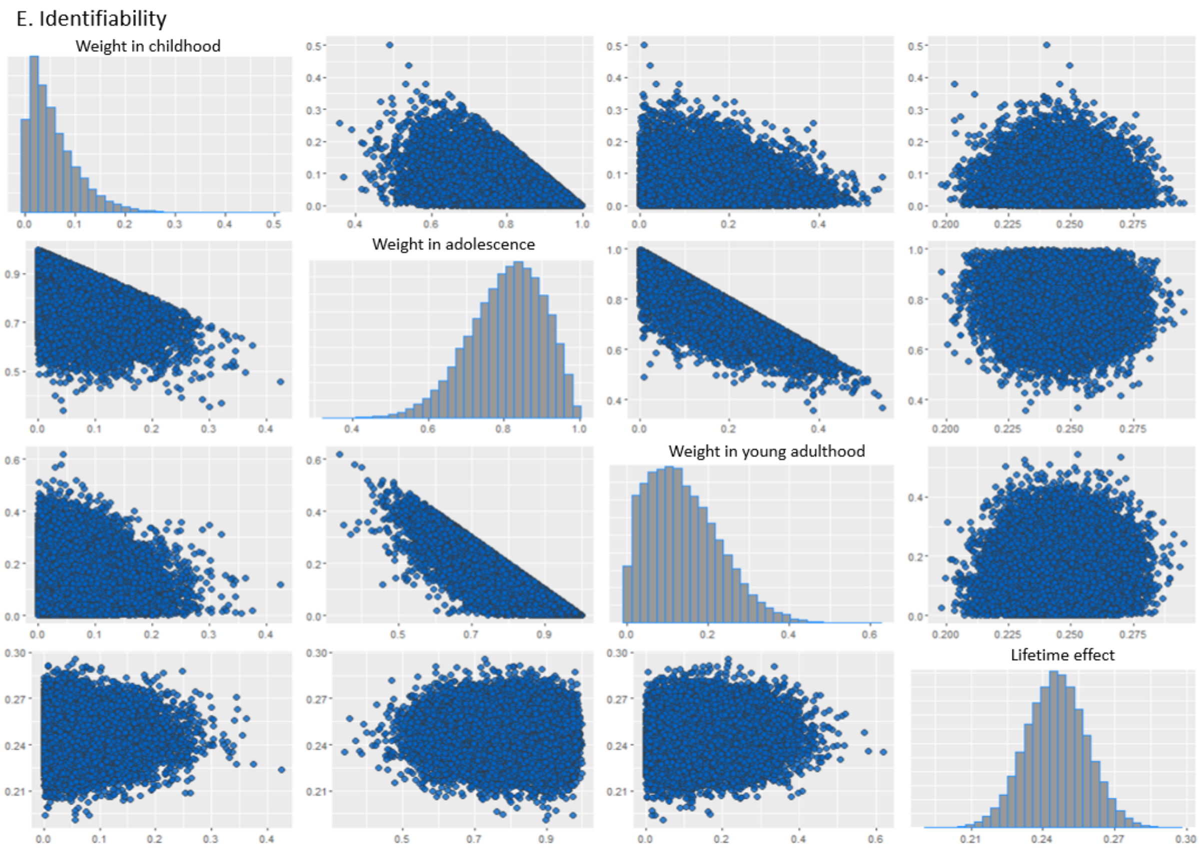

Figure S4. Diagnostics of the Bayesian relevant life-course exposure model, including (A) trace plots, (B) Rhat values, (C) effective sample size, (D) autocorrelation function plots, (E) identifiability of the parameters, for non-high-density lipoprotein cholesterol and carotid plaque areas in mid-adulthood aged 41 to 56 years in 2018. Missing covariates were imputed by multiple imputation, missing non-HDL-C data were interpolated by the Individual Growth Curve Model.

Comparison of posterior probabilities between life stages

All comparisons are based on the estimates derived from model 2 for each observed outcome variable.

(1) For the association of non-high-density lipoprotein cholesterol (non-HDL-C) and carotid plaque presence:

There is a 15 % posterior probability that the relative weight of non-HDL-C exposure in childhood is greater than the combined exposure in adolescence and young adulthood. There is a 25 % posterior probability that the relative weight of non-HDL-C exposure in adolescence is greater than the combined exposure in childhood and young adulthood. There is a 32 % posterior probability that the relative weight of non-HDL-C exposure in young adulthood is greater than the combined exposure in childhood and adolescence.

(2) For the association of non-HDL-C and carotid plaque area:

There is a 7 % posterior probability that the relative weight of non-HDL-C exposure in childhood is greater than the combined exposure in adolescence and young adulthood. There is a 55 % posterior probability that the relative weight of non-HDL-C exposure in adolescence is greater than the combined exposure in childhood and young adulthood. There is a 9 % posterior probability that the relative weight of non-HDL-C exposure in young adulthood is greater than the combined exposure in childhood and adolescence.

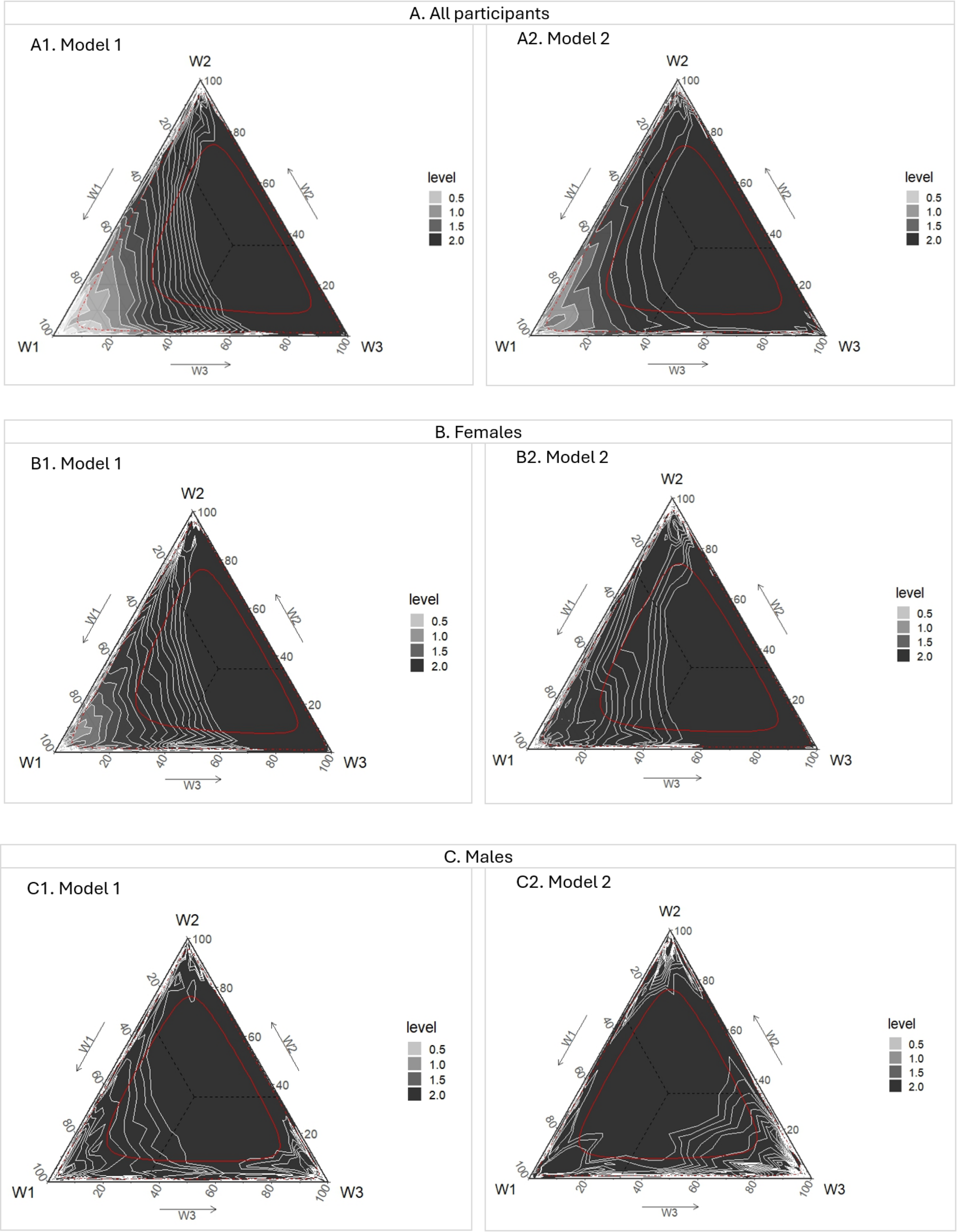

Figure S5. Posterior joint distribution of relative weights estimated for childhood, adolescence and young adulthood with the presence of carotid plaque, in A) all participants; B) females; C) males.

Abbreviations: W1, relative weight in childhood; W2, relative weight in adolescence; W3, relative weight in young adulthood.

Solid and dashed red lines represent 50 % and 95 % credible intervals.

Darker areas represent higher posterior densities.

Estimates derived from the Bayesian relevant life-course exposure models, in which non-high-density-lipoprotein cholesterol were used in 1-standard deviation scale. Model 1 adjusted for sex and year of birth. Model 2 adjusted for sex, year of birth, areas under curve between ages 6 to 24 years for body mass index, high-density-lipoprotein cholesterol and systolic blood pressure, areas under curve between ages 9 to 24 years for blood glucose and physical activity index, education (years studied), ever smoked daily before age 24 years, family history of cardiovascular disease in all participants.

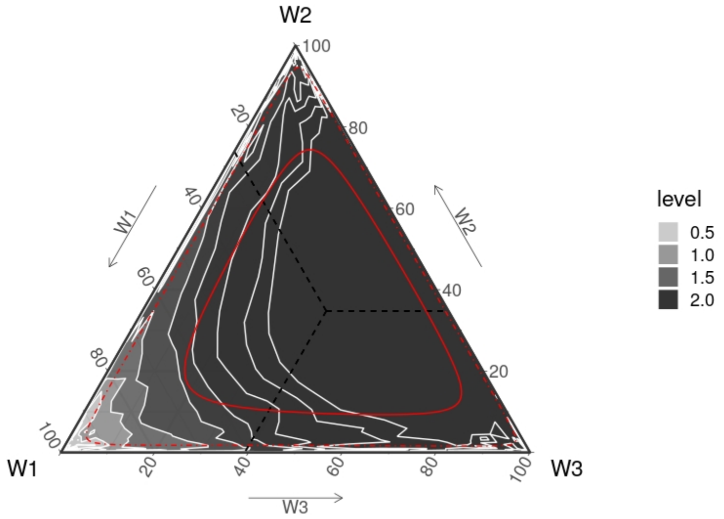

Figure S6. Posterior joint distribution of relative weights estimated for childhood, adolescence and young adulthood with the presence of carotid plaque.

Abbreviations: W1, relative weight in childhood; W2, relative weight in adolescence; W3, relative weight in young adulthood.

Solid and dashed red lines represent 50 % and 95 % credible intervals.

Darker areas represent higher posterior densities.

Estimates derived from the Bayesian relevant life-course exposure models, in which non-high-density-lipoprotein cholesterol were used in 1-standard deviation scale. Model adjusted for sex, year of birth, areas under curve between ages 6 to 24 years for body mass index, high-density-lipoprotein cholesterol and systolic blood pressure, areas under curve between ages 9 to 24 years for blood glucose and physical activity index, education (years studied), ever smoked daily before age 24 years, family history of cardiovascular disease in all participants. Missing covariates were imputed by multiple imputation, missing non-HDL-C data were interpolated by the Individual Growth Curve Model.

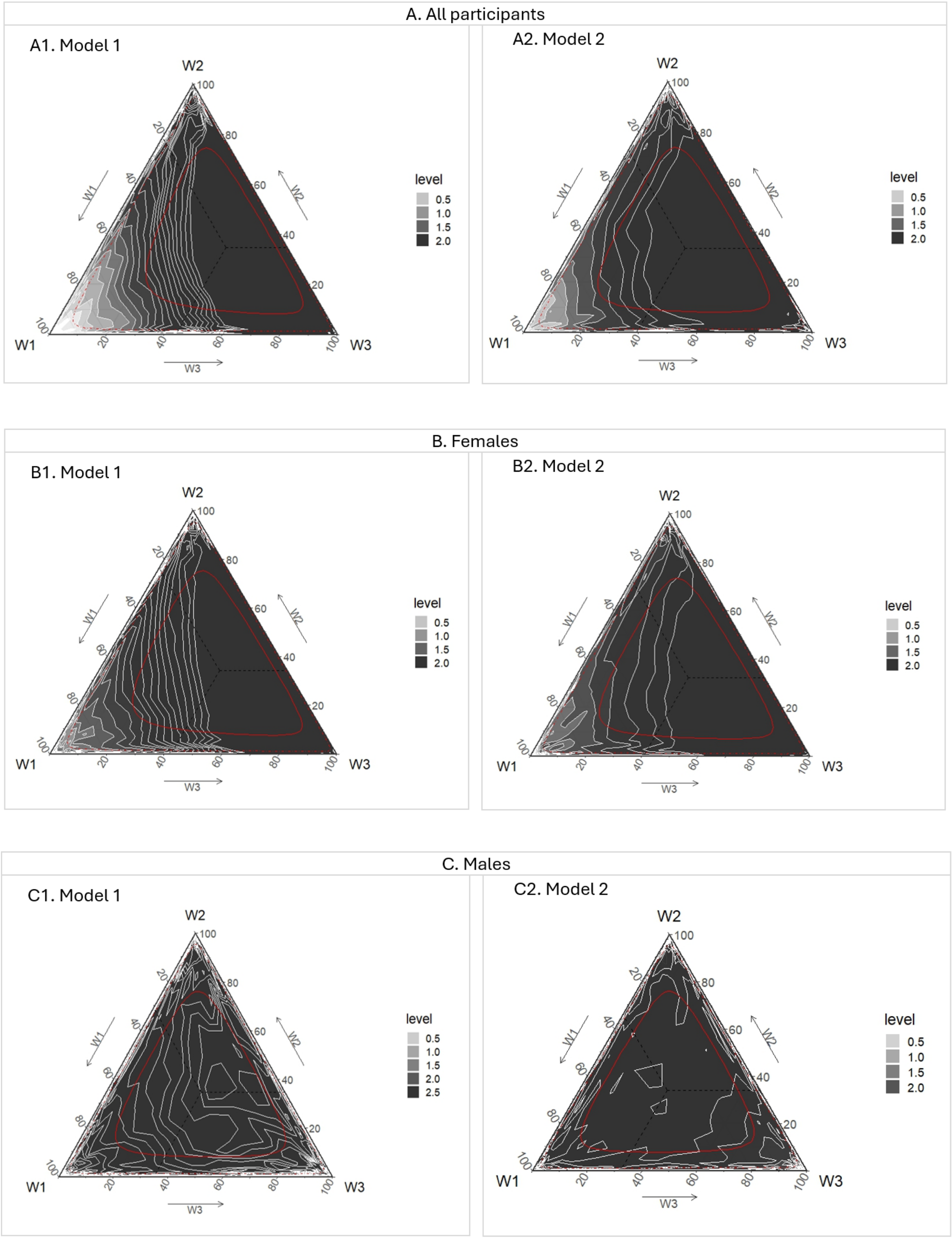

Figure S7. Posterior joint distribution of relative weights estimated for childhood, adolescence and young adulthood with the presence of carotid plaque, in A) all participants; B) females; C) males.

Abbreviations: W1, relative weight in childhood; W2, relative weight in adolescence; W3, relative weight in young adulthood.

Solid and dashed red lines represent 50 % and 95 % credible intervals.

Darker areas represent higher posterior densities.

Estimates derived from the Bayesian relevant life-course exposure models, in which non-high-density-lipoprotein cholesterol were used in 1-mmol/L scale. Model 1 adjusted for sex and year of birth. Model 2 adjusted for sex, year of birth, areas under curve between ages 6 to 24 years for body mass index, high-density-lipoprotein cholesterol and systolic blood pressure, areas under curve between ages 9 to 24 years for blood glucose and physical activity index, education (years studied), ever smoked daily before age 24 years, family history of cardiovascular disease in all participants.

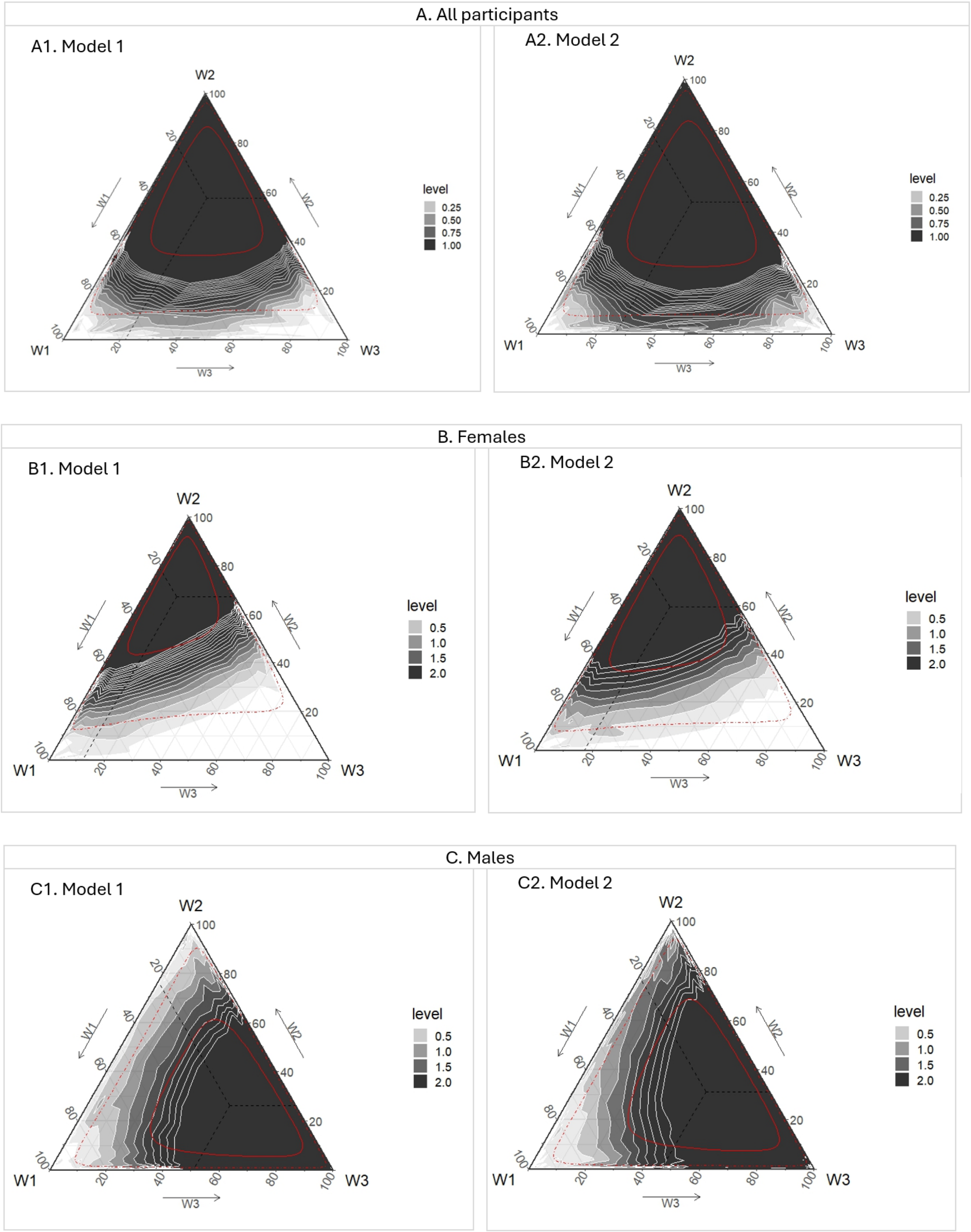

Figure S8. Posterior joint distribution of relative weights estimated for childhood, adolescence and young adulthood with carotid plaque areas, in A) all participants; B) females; C) males.

Abbreviations: W1, relative weight in childhood; W2, relative weight in adolescence; W3, relative weight in young adulthood.

Solid and dashed red lines represent 50 % and 95 % credible intervals.

Darker areas represent higher posterior densities.

Estimates derived from the Bayesian relevant life-course exposure models, in which non-high-density-lipoprotein cholesterol were used in 1-standard deviation scale. Model 1 adjusted for sex and year of birth. Model 2 adjusted for sex, year of birth, areas under curve between ages 6 to 24 years for body mass index, high-density-lipoprotein cholesterol and systolic blood pressure, areas under curve between ages 9 to 24 years for blood glucose and physical activity index, education (years studied), ever smoked daily before age 24 years, family history of cardiovascular disease in all participants.

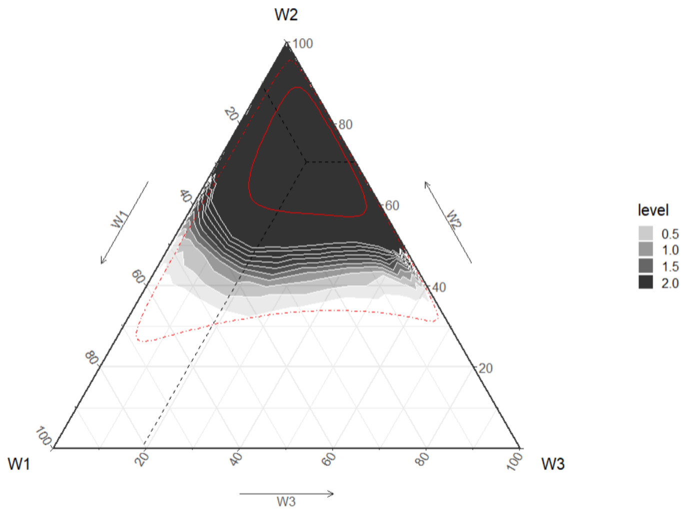

Figure S9. Posterior joint distribution of relative weights estimated for childhood, adolescence and young adulthood with carotid plaque areas

Abbreviations: W1, relative weight in childhood; W2, relative weight in adolescence; W3, relative weight in young adulthood.

Solid and dashed red lines represent 50 % and 95 % credible intervals.

Darker areas represent higher posterior densities.

Estimates derived from the Bayesian relevant life-course exposure models, in which non-high-density-lipoprotein cholesterol were used in 1-standard deviation scale. Model adjusted for sex, year of birth, areas under curve between ages 6 to 24 years for body mass index, high-density-lipoprotein cholesterol and systolic blood pressure, areas under curve between ages 9 to 24 years for blood glucose and physical activity index, education (years studied), ever smoked daily before age 24 years, family history of cardiovascular disease in all participants. Missing covariates were imputed by multiple imputation, missing non-HDL-C data were interpolated by the Individual Growth Curve Model.

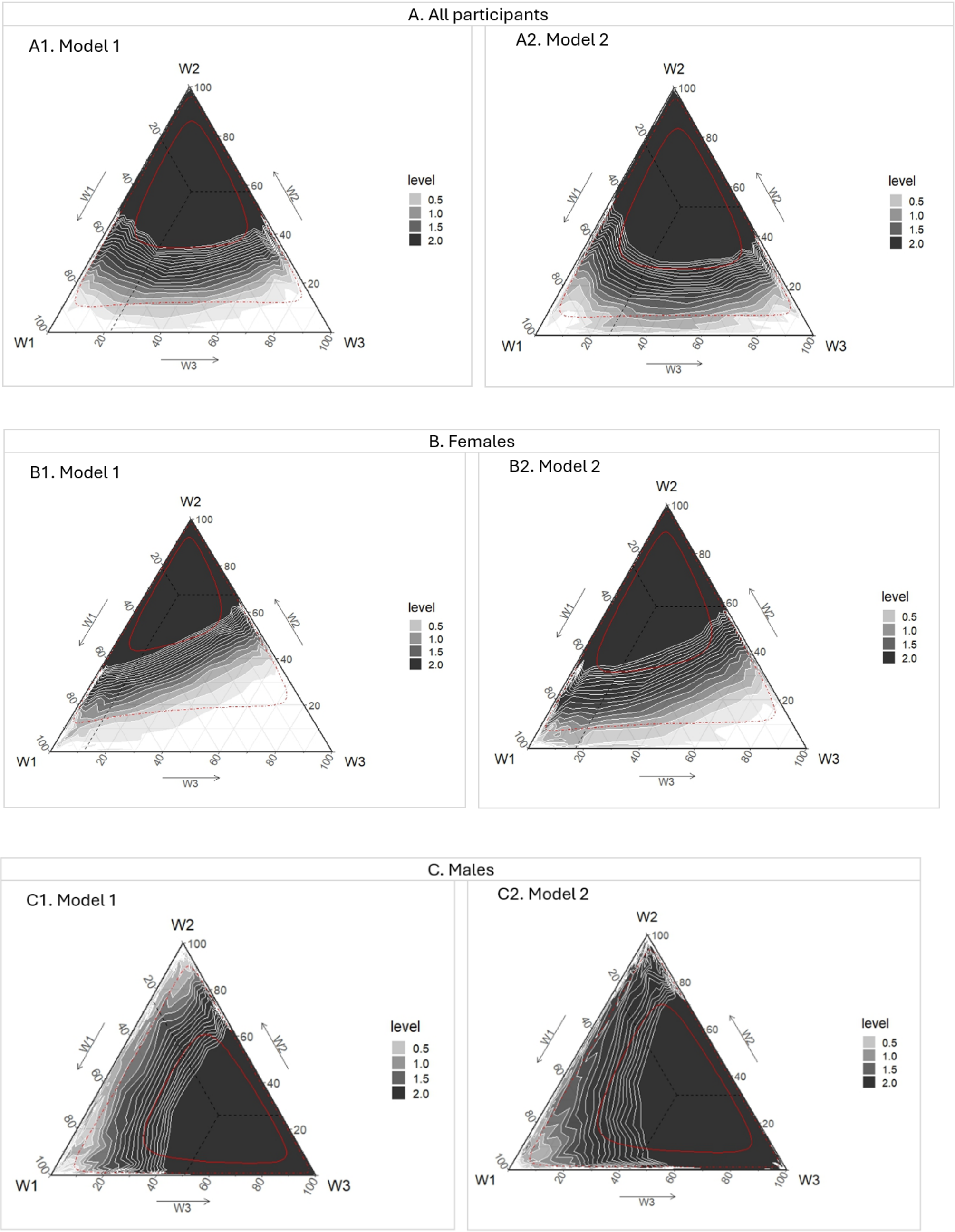

Figure S10. Posterior joint distribution of relative weights estimated for childhood, adolescence and young adulthood with carotid plaque areas, in A) all participants; B) females; C) males.

Abbreviations: W1, relative weight in childhood; W2, relative weight in adolescence; W3, relative weight in young adulthood.

Solid and dashed red lines represent 50 % and 95 % credible intervals.

Darker areas represent higher posterior densities.

Estimates derived from the Bayesian relevant life-course exposure models, in which non-high-density-lipoprotein cholesterol were used in 1-mmol/L scale. Model 1 adjusted for sex and year of birth. Model 2 adjusted for sex, year of birth, areas under curve between ages 6 to 24 years for body mass index, high-density-lipoprotein cholesterol and systolic blood pressure, areas under curve between ages 9 to 24 years for blood glucose and physical activity index, education (years studied), ever smoked daily before age 24 years, family history of cardiovascular disease in all participants.
