# Supplementary material for: Early intermittent hyperlipidaemia alters tissue macrophages to fuel atherosclerosis
Source: Nature. 2024 Sep 4;634(8033):457–65. doi: 10.1038/s41586-024-07993-x (PMC11464399; doi:10.1038/s41586-024-07993-x)
Supplement: Supplementary file 2 — Reporting Summary [file 41586_2024_7993_MOESM2_ESM.pdf]

nature portfolio

Corresponding author(s): Ziad Mallat

Last updated by author(s): Aug 9, 2024

Reporting Summary

Nature Portfolio wishes to improve the reproducibility of the work that we publish. This form provides structure for consistency and transparency in reporting. For further information on Nature Portfolio policies, see our Editorial Policies and the Editorial Policy Checklist.

Statistics

For all statistical analyses, confirm that the following items are present in the figure legend, table legend, main text, or Methods section.

n/a	Confirmed	
☐	☒	The exact sample size (*n*) for each experimental group/condition, given as a discrete number and unit of measurement
☐	☒	A statement on whether measurements were taken from distinct samples or whether the same sample was measured repeatedly
☐	☒	The statistical test(s) used AND whether they are one- or two-sided<br>*Only common tests should be described solely by name; describe more complex techniques in the Methods section.*
☐	☒	A description of all covariates tested
☐	☒	A description of any assumptions or corrections, such as tests of normality and adjustment for multiple comparisons
☐	☒	A full description of the statistical parameters including central tendency (e.g. means) or other basic estimates (e.g. regression coefficient) AND variation (e.g. standard deviation) or associated estimates of uncertainty (e.g. confidence intervals)
☐	☒	For null hypothesis testing, the test statistic (e.g. *F*, *t*, *r*) with confidence intervals, effect sizes, degrees of freedom and *P* value noted<br>*Give P values as exact values whenever suitable.*
☒	☐	For Bayesian analysis, information on the choice of priors and Markov chain Monte Carlo settings
☒	☐	For hierarchical and complex designs, identification of the appropriate level for tests and full reporting of outcomes
☒	☐	Estimates of effect sizes (e.g. Cohen's *d*, Pearson's *r*), indicating how they were calculated

Our web collection on statistics for biologists contains articles on many of the points above.

Software and code

Policy information about availability of computer code

Data collection	Reported in manuscript and in Supplementary Table 24
Data analysis	Reported in Methods section. Softwares used in the research are publicly available and are described in Supplementary Table 24. Code deposition released to publicly available with Github link https://github.com/CAD-ZM-BFX/Takaoka_Mallat (DOI: https://doi.org/10.5281/zenodo.13137366).

For manuscripts utilizing custom algorithms or software that are central to the research but not yet described in published literature, software must be made available to editors and reviewers. We strongly encourage code deposition in a community repository (e.g. GitHub). See the Nature Portfolio guidelines for submitting code & software for further information.

Data

Policy information about availability of data

All manuscripts must include a data availability statement. This statement should provide the following information, where applicable:

- Accession codes, unique identifiers, or web links for publicly available datasets
- A description of any restrictions on data availability
- For clinical datasets or third party data, please ensure that the statement adheres to our policy

All the associated raw data presented in this paper are available from the corresponding author upon reasonable request. Source data are provided with this paper.

Raw RNA-sequencing data is publicly accessible with accession number E-MTAB-12759 (https://www.ebi.ac.uk/biostudies/arrayexpress/E-MTAB-12759) and E-MTAB-12761(https://www.ebi.ac.uk/biostudies/arrayexpress/studies/E-MTAB-12761).

Human research participants

Policy information about studies involving human research participants and Sex and Gender in Research.

Reporting on sex and gender	Reported
Population characteristics	This is described in Methods and Supplementary Methods and patients characteristics are provided in Supplementary Tables 14 and 15. "Multiple imputation was undertaken for the covariates of youth age, ever smoked, family history of cardiovascular disease, education (number of years studied), and cumulative body mass index, systolic blood pressure and high-density cholesterol, using predictive mean matching via the R library, mice".
Recruitment	Described
Ethics oversight	Ethics approval for the baseline study was granted by the University of Turku Faculty of Medicine Ethical Committee (letter dated 14/11/1978, minutes 3/1978) and for the most recent follow-up, granted by the Hospital District of Southwest Finland (Ethics approval number ETMK:68/1801/2017).

Note that full information on the approval of the study protocol must also be provided in the manuscript.

Field-specific reporting

Please select the one below that is the best fit for your research. If you are not sure, read the appropriate sections before making your selection.

☒ Life sciences ☐ Behavioural & social sciences ☐ Ecological, evolutionary & environmental sciences

For a reference copy of the document with all sections, see nature.com/documents/nr-reporting-summary-flat.pdf

Life sciences study design

All studies must disclose on these points even when the disclosure is negative.

Sample size	We stated in the manuscript that "Sample size was determined to detect a significant difference (alpha=0.05 and 80% power) of at least 30% in lesion size between groups."
Data exclusions	No data exclusion
Replication	We indicated that "Main atherosclerosis experiments were repeated several times and showed consistent results (see Results)".
Randomization	We indicated that "Mice were randomly assigned to their respective experimental groups." The human data are derived from the Young Finns Study cohort and randomization is not applicable.
Blinding	For Atherosclerosis studies, we indicated that "Analyses were performed in a blinded manner". For staining quantification, we stated that "Data analysis was conducted in a blind manner where appropriate". For human carotid plaque measurements, we indicated that "Carotid artery plaque measures were performed off-line by a single reader blinded to participant's details".

Reporting for specific materials, systems and methods

We require information from authors about some types of materials, experimental systems and methods used in many studies. Here, indicate whether each material, system or method listed is relevant to your study. If you are not sure if a list item applies to your research, read the appropriate section before selecting a response.

Materials & experimental systems

n/a	Involved in the study	
☐	☒	Antibodies
☐	☒	Eukaryotic cell lines
☒	☐	Palaeontology and archaeology
☐	☒	Animals and other organisms
☒	☐	Clinical data
☒	☐	Dual use research of concern

Methods

n/a	Involved in the study	
☒	☐	ChIP-seq
☒	☐	Flow cytometry
☒	☐	MRI-based neuroimaging

Antibodies

Antibodies used	Antibodies are described in detail in Methods.<br>Primary mouse antibodies used for immunofluorescence analysis included anti-MOMA2 (rabbit monoclonal; Biorad MCA519G; 1:200), anti-CD68 (rat monoclonal; Bio-Rad MCA1957; 1:300), anti-CD206 (rat monoclonal; Bio-Rad MCA2235; 1:200), anti-CD3 (rabbit polyclonal, DAKO/Agilent A045229-2; 1:200), anti-LYVE-1 (rabbit monoclonal; Abcam ab218535; 1:600), anti-CD31 (Armenian-Hamster monoclonal; Millipore MAB1398Z; 1:200), anti-Collagen Type I (rabbit polyclonal; Millipore ABT257; 1:300), and Cy3-conjugated anti-smooth muscle actin (clone 1A4; Sigma-Aldrich C6198; 1:1000). CD45 (clone 30-F11, BioLegend, 0.5 ug/mL) and CD68 (clone FA-11, BioLegend, 1 ug/mL). Rat IgG (eBioscience) and rabbit IgG (Jackson ImmunoResearch) were used for isotype controls. AF488- (1:300), AF555- (1:300), Cy3- (1:500), AF647 (1:300)-conjugated antibodies (Jackson ImmunoResearch) were used for fluorescence detection.<br>Primary human antibodies were: CD68 (Agilent DAKO, M087601-2, Clone PG-M1, Monoclonal Mouse Anti-Human, 1:50);<br>Lyve1 (R&D Systems, AF2089, Polyclonal, Goat Anti-Human, 1:100).
Validation	Validation of antibodies was provided by the manufacturer, and this quality control data can be accessed through the supplier's website. In addition, all antibody clones have been widely used and tested in research with numerous prior publications validating their specificity.<br>Well-validated human antibodies were purchased from established commercial vendors, including Agilent, and, R&D Systems. Unless otherwise noted, antibodies were used at manufacturer- and primary literature-validated concentrations for the relevant assays, as detailed below:<br>CD68 (Agilent DAKO, M087601-2, Clone PG-M1, Monoclonal Mouse Anti-Human, 1:50): https://www.agilent.com/store/productDetail.jsp?catalogId=M087601-2&catId=SubCat3ECS_86401;<br>Lyve1 (R&D Systems, AF2089, Polyclonal, Goat Anti-Human, 1:100): https://www.rndsystems.com/products/human-lyve-1-antibody_af2089.<br>Well-validated mouse antibodies were purchased from established commercial vendors, including Bio-Rad, Merck Millipore, Abcam, Agilent DAKO and Sigma-Aldrich. Unless otherwise noted, antibodies were used at manufacturer- and primary literature-validated concentrations for the relevant assays, as detailed below:<br>MOMA2 (Monoclonal, Rat anti-mouse; Bio-Rad MCA519G, 1:200) https://www.bio-rad-antibodies.com/monoclonal/mouse-macrophages-monocytes-antibody-moma-2-mca519.html?f=purified<br>CD68 (Monoclonal, Rat anti-mouse; Bio-Rad MCA1957, 1:300) https://www.bio-rad-antibodies.com/monoclonal/mouse-cd68-antibody-fa-11-mca1957.html?f=purified<br>CD206 (Monoclonal, Rat anti-mouse ; Bio-Rad MCA2235, 1:200) https://www.bio-rad-antibodies.com/monoclonal/mouse-cd206-antibody-mr5d3-mca2235.html?f=purified<br>CD3 (Polyclonal, Rabbit anti-human/mouse DAKO/Agilent A045229-2, 1:200) https://www.agilent.com/store/en_US/Prod-A045229-2/A045229-2<br>LYVE-1 (Monoclonal, Rabbit anti-mouse; Abcam ab218535, 1:600) https://www.abcam.com/en-us/products/primary-antibodies/lyve1-antibody-epr21771-ab218535<br>CD31 (Monoclonal, Armenian-Hamster anti-mouse; Merck Millipore MAB1398Z, 1:200) https://www.merckmillipore.com/SG/en/product/Anti-PECAM-1-Antibody-clone-2H8-Azide-Free,MM_NF-MAB1398Z?ReferrerURL=https%3A%2F%2Fwww.google.com%2F<br>Collagen Type I (Polyclonal, Rabbit anti-mouse; Merck Millipore ABT257, 1:300) https://www.merckmillipore.com/SG/en/product/Anti-Pro-Collagen-Type-I-A1-COL1A1,MM_NF-ABT257<br>Smooth muscle actin (Monoclonal, Cy3-conjugated anti-mouse, clone 1A4; Sigma-Aldrich C6198, 1:1000) https://www.sigmaaldrich.com/SG/en/product/sigma/c6198?srsltid=AfmBOopf92OIh30s832a0Cvc6x3BCYiW3LEVIb_gxpzGwLQJ7bSM0Apn<br>CD45 (clone 30-F11, BioLegend, 0.5ug/mL; catalog #103126) and CD68 (clone FA-11, BioLegend, 1 ug/mL, catalog #137020).

Eukaryotic cell lines

Policy information about cell lines and Sex and Gender in Research

Cell line source(s)	Jurkat cells clone E6-1, ATCC.
Authentication	Jurkat cells have not been authenticated by our lab.
Mycoplasma contamination	The Jurkat cell line used in the experiment tested negative for mycoplasma at first provision. However, it has not been re-tested later on.
Commonly misidentified lines<br>(See ICLAC register)	n/a

Animals and other research organisms

Policy information about studies involving animals; ARRIVE guidelines recommended for reporting animal research, and Sex and Gender in Research

Laboratory animals	All mice were on a C57BL/6 background. Both males and females were used. Atherosclerosis experiments were started at the age of 6 weeks unless otherwise stated. Lyz2Cre+/−, Ldlr−/− and Rag2−/− mice were initially from Charles River. Spicflox/flox mice were from Tsuneyasu Kaisho, Department of Immunology, Institute of Advanced Medicine, Wakayama Medical University, Japan, and Dr. Wataru Ise, Laboratory of Lymphocyte Differentiation, World Premier International Immunology Frontier Research Center, Osaka University, Japan44. Lyz2Cre+/− Nrp1flox/flox and Lyz2Cre+/− Nrp1+/+ mice were generated by the group of Christiana Ruhrberg, University College London, UK. Cx3cr1creERT2/+ Rosa26LSL-Tomato Ldlr−/− mice were generated by Jesse Williams, University of Minnesota Medical School, Minneapolis, USA. Apoe-/- mice were purchased from Comparative Medicine (National University of Singapore). Lyve1cre/cre and Csf1rflox/flox mice were purchased from Jackson Laboratory. Apoe-/-Lyve1cre/wtCsf1rflox/flox mice

	were generated by crossbreeding Lyve1cre/cre with Apoe-/-Csf1rflox/flox mice.<br>Mice were housed in 12h/12h dark/light cycle, at ambient temperature of 22 degrees Celsius and 45%-65% humidity.
Wild animals	We indicated that "No wild animals and no field-collected samples were used in the study".
Reporting on sex	Reported. We indicated that "Both males and females were used". Sex is clearly identified in legends of figures and extended dat figures.
Field-collected samples	We indicated that "No wild animals and no field-collected samples were used in the study".
Ethics oversight	We stated that " All in vivo experiments using mice were approved by local Institutional Review Boards: the Home Office, UK, under PPL PA4BDF775; the Ethical Committee of INSERM (APAFIS #29371) and the French Ministry of Agriculture (MESRI 674 #29371); the Institutional Animal Care and Use Committee (IACUC) at National University of Singapore (protocol number R21-1562); and under animal protocol 2111-39587A for experiments performed in the USA".

Note that full information on the approval of the study protocol must also be provided in the manuscript.
